# Combined Treatment with Curcumin and Ferulic Acid Suppressed the Aβ-Induced Neurotoxicity More than Curcumin and Ferulic Acid Alone

**DOI:** 10.3390/ijms23179685

**Published:** 2022-08-26

**Authors:** Hideaki Ohashi, Mayumi Tsuji, Tatsunori Oguchi, Yutaro Momma, Tetsuhito Nohara, Naohito Ito, Ken Yamamoto, Miki Nagata, Atsushi Michael Kimura, Yuji Kiuchi, Kenjiro Ono

**Affiliations:** 1 Division of Medical Pharmacology, Department of Pharmacology, School of Medicine, Showa University, Tokyo 142-8555, Japan; 2 Division of Neurology, Department of Internal Medicine, School of Medicine, Showa University, Tokyo 142-8555, Japan; 3 Pharmacological Research Center, Showa University, Tokyo 142-8555, Japan; 4 Department of Hospital Pharmaceutics, School of Pharmacy, Showa University, Tokyo 142-8555, Japan; 5 Department of Neurology, Kanazawa University Graduate School of Medical Sciences, Kanazawa University, Kanazawa 920-8640, Japan

**Keywords:** amyloid-β, Alzheimer’s disease, curcumin, ferulic acid, oxidative stress, neurotoxicity

## Abstract

Alzheimer’s disease (AD) is a neurodegenerative disease that leads to progressive cognitive decline. Several effective natural components have been identified for the treatment of AD. However, it is difficult to obtain conclusive evidence on the safety and effectiveness of natural components, because a variety of factors are associated with the progression of AD pathology. We hypothesized that a therapeutic effect could be achieved by combining multiple ingredients with different efficacies. The purpose of this study was thus to evaluate a combination treatment of curcumin (Cur) and ferulic acid (FA) for amyloid-β (Aβ)-induced neuronal cytotoxicity. The effect of Cur or FA on Aβ aggregation using thioflavin T assay was confirmed to be inhibited in a concentration-dependent manner by Cur single or Cur + FA combination treatment. The effects of Cur + FA on the cytotoxicity of human neuroblastoma (SH-SY5Y) cells induced by Aβ exposure were an increase in cell viability, a decrease in ROS and mitochondrial ROS, and repair of membrane damage. Combination treatment showed an overall higher protective effect than treatment with Cur or FA alone. These results suggest that the combined action mechanisms of Cur and FA may be effective in preventing and suppressing the progression of AD.

## 1. Introduction

Alzheimer’s disease (AD), an age-related neurodegenerative disorder that causes progressive cognitive decline, represents the most frequent form of dementia. The “Alzheimer’s Association Report 2021” predicts that more than 152 million people will have AD by 2050 [1]. One of the neuropathological features of AD is the deposition of senile plaques and neurofibrillary changes in the brain. The significant components of senile plaques and neurofibrillary changes have been identified as amyloid-β protein (Aβ) and highly phosphorylated tau protein. These protein aggregates induce neuronal damage and cell death, resulting in memory and learning disabilities that lead to dementia. The exact cause of AD is not yet clear, but a number of studies have shown that oxidative stress, mitochondrial dysfunction, and inflammation play a major role in its onset and progression [2]. The amyloid cascade hypothesis has also been a major theoretical component of research on AD for over 20 years [3]. Importantly, Aβ begins to accumulate in the brain 10 to 20 years or more before the cognitive decline is observed [4], and there is therefore an urgent need to develop therapeutic agents that suppress the accumulation of Aβ from the preclinical stage when the brain is not yet severely damaged [5].

The currently widely used therapeutic drugs for AD are acetylcholinesterase inhibitors and N-methyl-D-aspartic acid (NMDA) receptor antagonists, which are intended to temporarily slow the progression of symptoms; they are, however, not the ultimate treatment. What is needed is a therapeutic approach that can prevent or delay the progression of AD. In recent years, interest in low-molecular-weight natural products that are inexpensive, easy to consume, and have multiple physiological effects has been increasing. In particular, polyphenols, which are plant components, exert potent antioxidant and physiological effects on, for example, blood flow, obesity, and blood pressure [6]. Polyphenols are thought to be neuroprotective, because of their ability to influence pathways associated with the pathogenesis of AD [7].

Among these, curcumin (Cur) has been studied for its antioxidant and anti-inflammatory effects [8]. Cur is a fat-soluble polyphenol abundant in turmeric, traditionally used in Indian medicine. In some in vivo and in vitro studies, Cur has been found to have antioxidant and anti-inflammatory effects, and it is therefore attracting attention as a preventative action for AD by inhibiting Aβ aggregation including oligomerization in vitro and in vivo [9,10]. Furthermore, the administration of Cur to APPswe/PS1dE9 double transgenic mice has been found to reduce the γ-secretase component presenilin-2 and promote the degradation of aggregated Aβ [11]. However, clinical trials on the effects of Cur in AD have provided inconsistent results that are difficult to interpret [12]. One possible reason for these inconsistencies may be that Cur is poorly absorbed and rapidly metabolized and excreted, which results in low bioavailability due to low plasma and tissue levels [13]. To enhance the low bioavailability of Cur, a variety of approaches have been taken, such as combining it with conventional drugs and developing new delivery systems.

Ferulic acid (FA) is a phenolic compound found abundantly in plant cell walls, for example in Ranunculaceae and Gramineae, and is known as a biologically active compound with multiple pharmacological functions, including antioxidant [14], anti-inflammatory [15], and anticancer effects [16]. In addition, FA has been shown to be protective against cardiovascular disease [17] and prevent coronary heart disease [18] and atherosclerosis [19]. Moreover, FA has also been reported to reduce Aβ deposition and IL-1β levels in the frontal cortex and improve performance on novel-object recognition tasks in APPswe/PS1dE9 double transgenic mice [20]. Actually, we previously confirmed that FA dose-dependently not only inhibited Aβ fibril formation, but also destabilized preformed fibrils [21]. PSAPP mice treated with FA exhibited reduced Aβ deposition in the parenchyma and cerebral blood vessels [22].

Cur and FA share some similarities, and Cur is metabolized to FA by the cleavage reaction. FA has better bioavailability and metabolic stability than Cur. However, previous studies of AD have not established a definitive treatment with Cur or FA. The development of therapeutic agents in AD may benefit from a multi-targeted approach, including the inhibition of Aβ aggregation and neuronal protection. For substances that act on many targets, the simultaneous administration of multiple compounds with more different biological activities is considered more effective than a single agent. Treatment with a combination of compounds may also reduce the required concentration of each compound and lower the risk of side effects.

**Figure 1:**
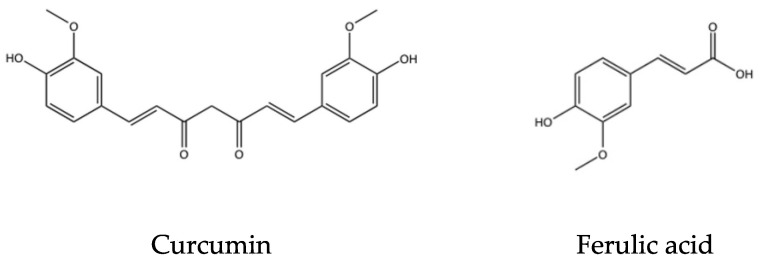
Structures of curcumin and ferulic acid.

In this study, we compared the protective effects of Cur and FA (Figure 1), which differ in their major mechanism of Aβ-induced neurotoxicity, with the protective effects of combined treatment with both compounds. Our results show that combination treatment with Cur and FA has significant advantages over single treatment with either compound.

## 2. Results

### 2.1. Effects of Cur, FA, and Their Combination on the Aggregation of Aβ_1–40_ and Aβ_1–42_

**Figure 2:**
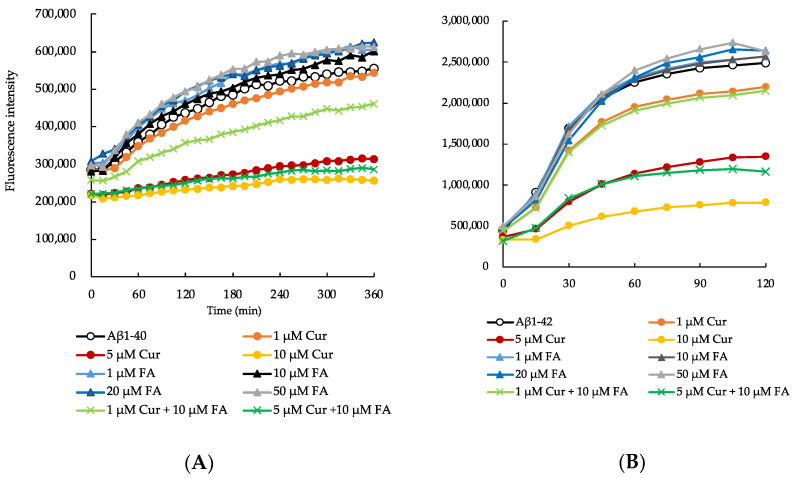
The aggregation kinetics of Aβ_1–40_ (**A**) and Aβ_1–42_ (**B**) with Cur, FA, or Cur + FA as measured with the ThT assay.

We compared the effects of Cur, FA, and the combination of both on the aggregation kinetics of Aβ_1–40_ and Aβ_1–42_ peptides using a thioflavin T (ThT) fluorescence assay. First, we monitored the amyloid formation of Aβ_1–40_ (25 μM) (Figure 2A,B). The aggregation of the Aβ_1–40_ peptide alone increased exponentially with no delay and reached approximately twice the fluorescence intensity observed at onset after 6 h. However, in the presence of Cur, the fluorescence intensity of ThT decreased in a concentration-dependent manner as compared to peptide incubated alone, with 10 μM Cur instigating an inhibition of 55% after 360 min (Aβ_1–40_ alone: 554,963.3 ± 1783.8, 1 μM Cur: 543,495 ± 4097.2, 5 μM Cur: 314,051 ± 3105.8, 10 μM Cur: 255,990.8 ± 11061.9, mean ± S.E.M, n = 6) (Figure 2A). However, there was no marked effect on Aβ_1–40_ aggregation in the presence of FA (1 μM FA: 603,630 ± 4366, 10 μM FA: 601,316.8 ± 2074.1, 20 μM FA: 624,204.3 ± 5418.4, 50 μM FA: 612,349.7 ± 3175.4). In combination of Cur and FA, the fluorescence of Aβ_1–40_ incubated with combination was significantly lower than that in the presence of 10 μM FA at 360 min (1 μM Cur + 10 μM FA: 461,661.3 ± 5780.2, 5 μM Cur + 10 μM FA: 286,174 ± 6329.2, n = 6) (Figure 2A and Table S1).

Next, we monitored the aggregation kinetics of Aβ_1–42_ peptide for 120 min (Figure 2B). The aggregation of Aβ_1–42_ peptide causes significant neurotoxicity among all existing isoforms of Aβ [23]. Therefore, we also investigated the inhibitory effects of Cur, FA, and the combination of both on Aβ_1–42_ aggregation kinetics, as described above for Aβ_1–40_. The aggregation rate of Aβ_1–42_ peptide was much faster than that of Aβ_1–40_ peptide. In a previous study, we had also observed that the aggregation kinetics of Aβ_1–42_ peptide alone showed an exponential increase without any lag phase [24]. Here, the first stage was an exponential increase in ThT fluorescence intensity, after which the stationary phase was reached. The maximum saturation fluorescence intensity value of the stationary phase increased to about 5.5-fold the fluorescence intensity at onset. Similar to the aggregation kinetics of the Aβ_1–40_ peptide, we observed that the incubation of Aβ_1–42_ peptide with Cur resulted in a concentration-dependent decrease in fluorescence intensity. After 120 min, the fluorescence of Aβ_1–42_ incubated with concentration above 1 μM Cur was significantly lower than that in the absence of Cur (Aβ_1–42_ alone: 2,518,582.3 ± 27,558.1, 1 μM Cur: 2,196,175.5 ± 32,443.2, 5 μM Cur: 1,349,023 ± 15908.3, 10 μM Cur: 788,601.5 ± 66,620.7, n = 6) (Figure 2B and Table S2). Around 70% inhibition of Aβ_1–42_ peptide aggregation was observed after incubation with 10 μM Cur for 120 min. However, similar to Aβ_1–40_, no significant effect on Aβ_1–42_ aggregation was observed in the presence of FA (1 μM FA: 2,568,661 ± 48,401.9, 10 μM FA: 2,568,768.5 ± 15,582.4, 20 μM FA: 2,641,045.4 ± 54,318.4, 50 μM FA: 612,349.7 ± 3175.4). With the combination of Cur and FA, the fluorescence of Aβ_1–42_ incubated with the combination was significantly lower than that in the presence of 10 μM FA at 120 min (1 μM Cur + 10 μM FA: 2,150,414.8 ± 7235.5, 5 μM Cur + 10 μM FA: 1,164,776.6 ± 58,882, n = 6) (Figure 2B and Table S2).

Cur, FA or Cur + FA containing 25 µM Aβ (_1–40_ or _1–42_) with 200 µM ThT was incubated at 37 °C. Aβ_1–40_ was used in the ThT assay in the presence of Cur (1, 5, 10 μM), FA (1, 10, 20, 50 μM), 1 µM Cur + 10 µM FA and 5 µM Cur + 10 µM FA. 

### 2.2. Effects of Cur, FA, and Their Combination on Viability and Neurotoxicity in Aβ_1–42_-Exposed Cells

#### 2.2.1. Changes in Viability Assessed with MTT (3-(4,5-Dimethylthiazol-2-yl)-2,5-Diphenyltetrazolium Bromide)

**Figure 3:**
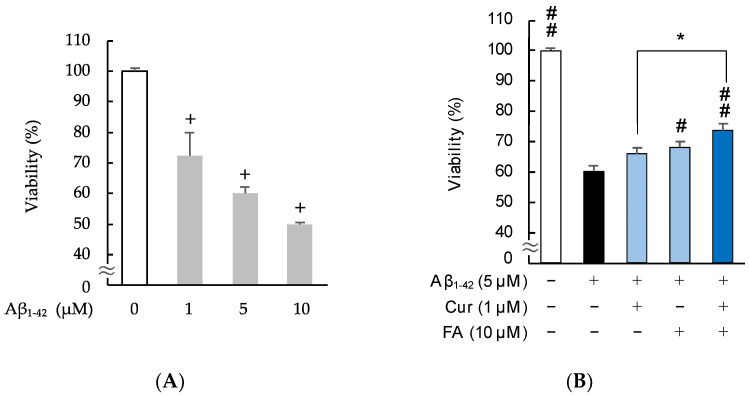
Effect of Cur, FA, or a combination of both on the viability in Aβ_1–42_-stimulated SH-SY5Y cells. The viability in Aβ_1–42_-stimulated SH-SY5Y cells was evaluated using MTT assay. (**A**) Cell viability of SH-SY5Y cells exposed to 5 µM Aβ_1–42_ (1, 5 and 10 µM) for 3 h. (**B**) Cell viability of SH-SY5Y cells exposed to 5 µM Aβ_1–42_ and treated with Aβ_1–42_ + 1 µM Cur, Aβ_1–42_ + 10 µM FA or Aβ_1–42_ + Cur + FA for 3 h. +: inclusion of 5 μM Aβ_1__–– 42_, 1 μM Cur, 10 μM FA, respectively, −: non-inclusion. The *p*-values in ANOVA were < 0.001. Each value expresses the mean + S.E.M. of at least 3 independent experiments. In the absence of 5 μM Aβ_1–42_, viabilities of control, 1 μM Cur, 10 μM FA and Cur + FA-treated cells were 100.0 ± 1.081, 102.2 ± 2.85, 98.6 ± 2.20 and 103.4 ± 2.00% (no significant difference, n = 6, Tukey). +, *p* < 0.01 for control versus Aβ_1–42_ exposed cells (n = 6, Dunnet’s); #, *p* < 0.05; ##, *p* < 0.01 for Aβ_1–42_ exposed cells versus the other treated cells (n = 6, Tukey); * *p* < 0.05 for Aβ_1–42_ + Cur + FA-treated cells versus Cur-treated cells (n = 6, Tukey).

As shown in Figure 3 and Figure S1, to determine the neurotoxicity of Aβ_1–42_ and to compare the effects of Cur, FA, and their combination on cells, the viability of SH-SY5Y cells was evaluated at 3 h after treatment. The results of an MTT assay revealed that exposure of SH-SY5Y cells to Aβ_1–42_ for 3 h significantly reduced cell viability in a concentration-dependent manner (Figure 3A). Based on these findings, we decided to examine neurotoxicity after exposure to 5 μM Aβ_1–42_ in SH-SY5Y cells. Viability significantly decreased with the 5 μM Aβ_1–42_ exposure, and the decrease was significantly recovered by treatment with 10 μM FA (*p* = 0.0135 vs. 5 μM Aβ_1–42_), and the combination of both (*p* < 0.0001 vs. 5 μM Aβ_1–42_). Moreover, the viability of cells treated with the combination of 1 uM Cur + 10 uM FA was increased compared to cells treated with 1 μM Cur alone (n = 6, Tukey, *p* = 0.0488).

#### 2.2.2. Staining with Calcein-AM/Ethidium Homodimer-1 (EthD-1)

**Figure 4:**
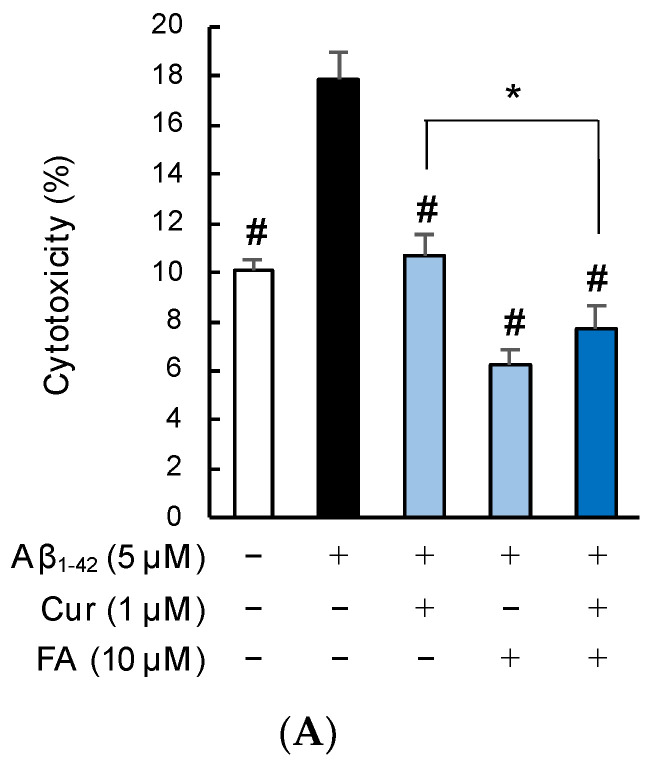

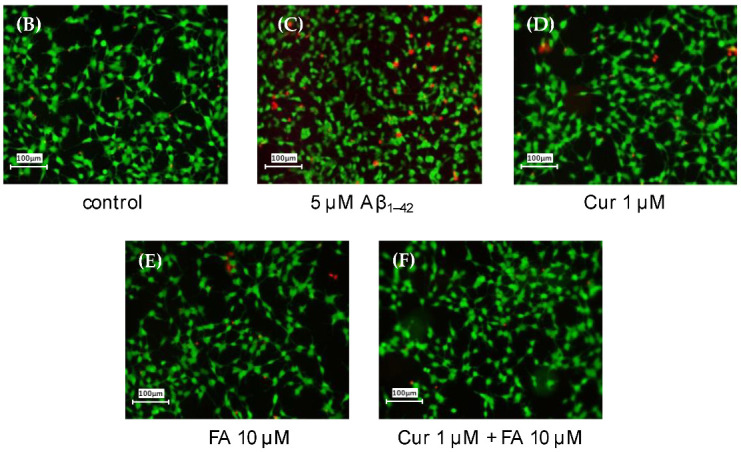
Effect of Cur, FA, or a combination of both on the cytotoxicity in Aβ_1–42_-stimulated SH-SY5Y cells. The cytotoxicity in Aβ_1–42_-stimulated SH-SY5Y cells was evaluated using EthD-1 Cell assay. (**A**) The cytotoxicity of SH-SY5Y cells exposed to 5 µM Aβ_1–42_ and treated with Aβ_1–42_ + 1 µM Cur, Aβ_1–42_ + 10 µM FA or Aβ_1–42_ + 1 µM Cur + FA. (**B**–**E**) Fluorescence microscopic images in SH-SY5Y cells were acquired using an inverted fluorescence microscope. (**B**) Untreated SH-SY5Y cells; (**C**) SH-SY5Y cells exposed to 5 μM Aβ_1–42_; (**D**) SH-SY5Y cells treated with 5 μM Aβ_1–42_ + 1 μM Cur; (**E**) SH-SY5Y cells treated with 5 μM Aβ_1–42_ + 10 μM FA; (**F**) SH-SY5Y cells treated with 5 μM Aβ_1–42_ + 1 μM Cur + 10 μM FA. The scale bar represents 100 µm. +: inclusion of 5 μM Aβ_1__–– 42_, 1 μM Cur, 10 μM FA, respectively, −: non-inclusion. The *p*-values in ANOVA were < 0.001. Each value expresses the mean + S.E.M. of at least 3 independent experiments. In the absence of 5 μM Aβ_1–42_, cytotoxicity of control, 1 μM Cur, 10 μM FA and Cur + FA-treated cells were 10.48 ± 0.52, 10.21± 1.54, 10.27 ± 2.06 and 10.30 ± 1.73% (no significant difference, n = 6, Tukey). #, *p* < 0.0001 for Aβ_1–42_ exposed cells versus the other treated cells (n = 6, Tukey); *, *p* <0.05 for Aβ_1–42_ + Cur + FA-treated cells versus Cur-treated cells (n = 6, Tukey).

Figure 4 shows the results of calcein-AM/EthD-1 staining of SH-SY5Y cells incubated with 5 μM Aβ_1–42_ for 3 h in the presence of Cur, FA, or a combination of both. Cell cytotoxicity was significantly increased in cells exposed to Aβ_1–42_ alone compared with control cells, while cell cytotoxicity induced by Aβ_1–42_ was remarkably suppressed by treatment with 1 μM Cur (*p* < 0.0001 vs. 5 μM Aβ_1–42_), 10 μM FA (*p* < 0.0001 vs. 5 μM Aβ_1–42_), or both (*p* < 0.0001 vs. 5 μM Aβ_1–42_) for 3 h (n = 6, Tukey). The combination of 1 μM Cur + 10 μM FA (*p* = 0.0461 vs. 1 μM Cur) significantly reduced cytotoxicity levels compared to treatment with 1 μM Cur alone (Figure 4A). When the SH-SY5Y cells were assessed with a fluorescence microscope, red fluorescence was observed in damaged cells exposed to Aβ_1–42_ and, in comparison, diminished in cells treated with Cur, FA, or Cur + FA (Figure 4B–F).

### 2.3. Oxidative Stress

Since Aβ_1–42_ induces oxidative stress, oxidative stress is associated with Aβ_1–42_ [25]. It has been suggested that oxidative stress plays an important role in the pathogenesis of AD because increased oxidative stress contributes to cell membrane damage and cell death. Then, the protective effects of Cur, FA and the combination of both compounds on Aβ_1–42_-induced oxidative stress were investigated.

#### 2.3.1. ROS Production

**Figure 5:**
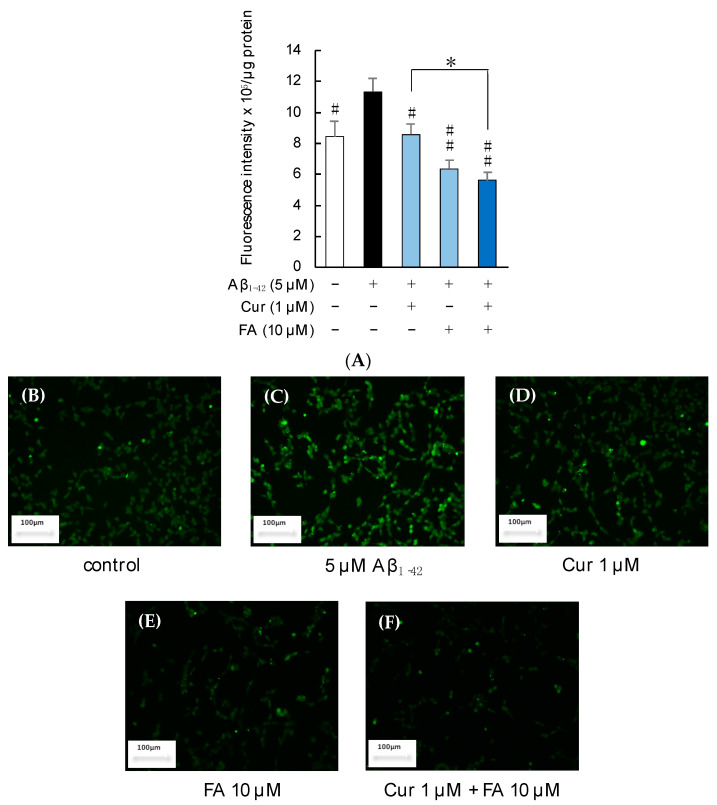
Effect of Cur, FA, or a combination of both on ROS generation in Aβ_1–42_-stimulated SH-SY5Y cells. The generation of ROS in Aβ_1–42_-stimulated SH-SY5Y cells was evaluated using 2′,7′-dichlorodihydrofluorescein diacetate (CM-H_2_DCFDA). (**A**) The generation of ROS in SH-SY5Y cells exposed to 5 µM Aβ_1–42_ and treated with Aβ_1–42_ + 1 µM Cur, Aβ_1–42_ + 10 µM FA or Aβ_1–42_ + Cur + FA. (**B**–**F**) Fluorescence microscopic images in SH-SY5Y cells were acquired using an inverted fluorescence microscope. (**B**) Untreated SH-SY5Y cells; (**C**) SH-SY5Y cells exposed to 5 μM Aβ_1–42_; (**D**) SH-SY5Y cells treated with 5 μM Aβ_1–42_ + 1 μM Cur; (**E**) SH-SY5Y cells treated with 5 μM Aβ_1–42_ + 10 μM FA; (**F**) SH-SY5Y cells treated with 5 μM Aβ_1–42_ + 1 μM Cur + 10 μM FA. The scale bar represents 100 µm. In the absence of 5 μM Aβ_1–42_, ROS levels of control, 1 μM Cur, 10 μM FA and Cur + FA-treated cells were 8.19 ± 0.97, 7.74 ± 0.55, 7.64 ± 0.12 and 7.51 ± 0.17 fluorescence intensity x 10^6^/μg protein (no significant difference, n = 6, Tukey). +: inclusion of 5 μM Aβ_1__–– 42_, 1 μM Cur, 10 μM FA, respectively, −: non-inclusion. The *p*-values for ANOVA were <0.001. Each value expresses the mean + S.E.M. of at least 3 independent experiments. #, *p* < 0.05; ##, *p* < 0.001 for Aβ_1–42_ exposed cells versus the other treated cells (n = 6, Tukey); *, *p* < 0.05 for Aβ_1–42_ + Cur + FA-treated cells versus Cur-treated cells (n = 6, Tukey).

Figure 5 shows the levels of ROS production in SH-SY5Y cells incubated with 5 μM Aβ_1–42_ for 30 min in the presence of Cur, FA, or the combination of both. ROS production significantly increased in SH-SY5Y cells exposed to Aβ_1–42_ (5 µM) compared with control cells. However, the increase due to Aβ_1–42_ exposure was significantly suppressed after 30 min of treatment with 1 µM Cur (*p* = 0.0466 vs. 5 µM Aβ_1–42_), 10 µM FA (*p* = 0.0003 vs. 5 µM Aβ_1–42_), or the combination of both (*p* < 0.0001 vs. 5 µM Aβ_1–42_). In particular, treatment with the combination of 1 µM Cur and 10 µM FA significantly decreased ROS production, compared to treatment with 1 μM Cur alone (Tukey, *p* = 0.0499) (Figure 5A). In addition, the combination treatment of 5 μM Cur and 10 μM FA significantly reduced ROS production, compared to 5 μM Cur single treatment (Tukey, *p* = 0.0057) (Figure S2A). The images obtained with a fluorescence microscope are shown in Figure 5B–F. The fluorescence of green dichlorofluorescein (DCF) was enhanced by 5 μM Aβ_1–42_ exposure (Figure 5C) but the green fluorescence was reduced by treatment with Cur, FA, or the combination of both (Figure 5D–F).

#### 2.3.2. Mitochondrial ROS Production and Manganese Superoxide Dismutase (Mn-SOD) Levels

**Figure 6:**
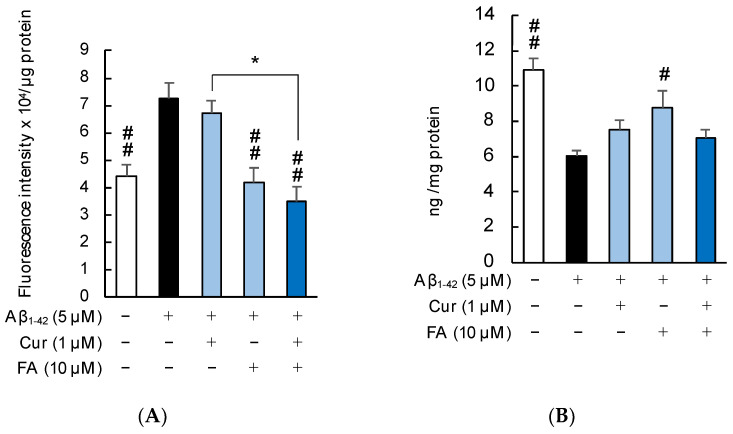
Effect of Cur, FA, or a combination of both on mitochondrial ROS and Mn-SOD in Aβ_1–42_-stimulated SH-SY5Y cells. Mitochondrial ROS levels were measured using a mitochondrial ROS detection reagent. Mn-SOD levels were measured using the Human SOD2 ELISA Kit. (**A**) The levels of mitochondrial ROS in SH-SY5Y cells exposed to 5 µM Aβ_1–42_ and treated with Aβ_1–42_ + 1 µM Cur, Aβ_1–42_ + 10 µM FA or Aβ_1–42_ + Cur + FA. In the absence of 5 μM Aβ_1–42_, mitochondrial ROS levels of control, 1 μM Cur, 10 μM FA and Cur + FA-treated cells were 4.26 ± 0.23, 4.38 ± 0.47, 4.52 ± 0.66 and 4.05 ± 0.42 x 10^4^/μg protein (no significant difference, n = 6, Tukey). (**B**) The concentration of Mn-SOD in SH-SY5Y cells exposed to 5 µM Aβ_1–42_ and treated with Aβ_1–42_ + 1 µM Cur, Aβ_1–42_ + 10 µM FA or Aβ_1–42_ + Cur + FA. In the absence of 5 μM Aβ_1–42_, Mn-SOD levels of control, 1 μM Cur, 10 μM FA and Cur + FA-treated cells were 10.83 ± 0.79, 10.47 ± 1.21, 10.23 ± 1.14 and 10.20 ± 0.50 ng/mg protein (no significant difference, n = 6, Tukey). +: inclusion of 5 μM Aβ_1__–– 42_, 1 μM Cur, 10 μM FA, respectively, −: non-inclusion. The *p*-values in ANOVA were <0.001. Each value expresses the mean + S.E.M. of at least 3 independent experiments. #, *p* < 0.05; ##, *p* < 0.01 for Aβ_1–42_ exposed cells versus the other treated cells (n = 6, Tukey): *, *p* <0.01 for Aβ_1–42_ + Cur + FA-treated cells versus Cur-treated cells (n = 6, Tukey).

Figure 6 shows the levels of mitochondrial ROS and Mn-SOD production in SH-SY5Y cells incubated with 5 μM Aβ_1–42_ in the presence of Cur, FA, or combinations of both. As shown in Figure 6A, levels of mitochondrial ROS production were significantly increased by 5 μM Aβ_1–42_ exposure and significantly suppressed by treatment with FA (*p* = 0.0019 vs. 5 μM Aβ_1–42_) or combination of Cur + FA (*p* < 0.0001 vs. 5 μM Aβ_1–42_). Combination treatment with Cur and FA significantly reduced mitochondrial ROS production compared to treatment with Cur alone (Tukey, *p* = 0.001) (Figure 6A).

Superoxide dismutase has the role of protecting cells from ROS by dismutation of superoxide radicals to molecular oxygen and hydrogen peroxide. As shown in Figure 6B, Mn-SOD levels were significantly decreased in SH-SY5Y cells after Aβ_1–42_ exposure, which was reversed by treatment with 10 μM FA (*p* = 0.0197 vs. 5 μM Aβ_1–42_), but had no effect on Cur + FA treatment. Moreover, there was no marked effect on Mn-SOD levels in treated cells with their combination compared to Cur or FA alone.

### 2.4. Effects of Cur or FA and Their Combination on Aβ_1–42_-Induced Disruption of Membrane Integrity

Aβ_1–42_ is thought to bind directly to membrane lipids, damage the phospholipid bilayer structure, and invade cells [26,27]. In this study, changes in cell membrane fluidity and cell membrane phospholipid peroxidation due to Aβ exposure were investigated.

#### 2.4.1. Fluidity of the Cell Membrane

**Figure 7:**
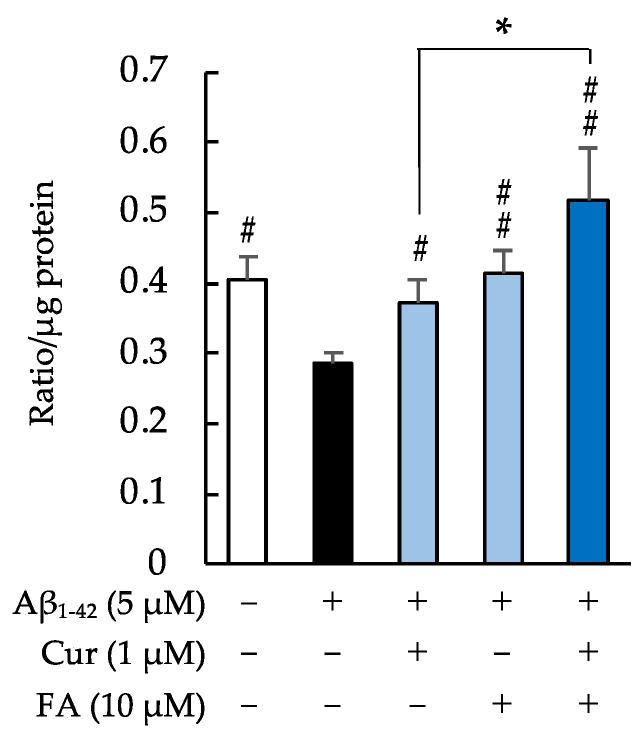
Effect of Cur, FA, or a combination of both on cell membrane fluidity Aβ_1–42_-stimulated SH-SY5Y cells. The fluidity of cell membrane in Aβ_1–42_-stimulated SH-SY5Y cells was evaluated using PDA. The fluidity of cell membrane in SH-SY5Y cells exposed to 5 µM Aβ_1–42_ and treated with Aβ_1–42_ + 1 µM Cur, Aβ_1–42_ + 10 µM FA or Aβ_1–42_ + Cur + FA. In the absence of 5 μM Aβ_1–42_, membrane fluidity levels of control, 1 μM Cur, 10 μM FA and Cur + FA-treated cells were 0.41 ± 0.045, 0.38 ± 0.036, 0.42 ± 0.041 and 0.43 ± 0.064 ratio/μg protein (no significant difference, n = 6, Tukey). +: inclusion of 5 μM Aβ_1__–– 42_, 1 μM Cur, 10 μM FA, respectively, −: non-inclusion. The *p*-values in ANOVA were <0.001. Each value expresses the mean + S.E.M. of at least 3 independent experiments. #, *p* < 0.05; ##, *p* < 0.001 for Aβ_1–42_ exposed cells versus the other treated cells (n = 6, Tukey); *, *p* < 0.05; for Aβ_1–42_ + Cur + FA-treated cells versus Cur-treated cells (n = 6, Tukey).

The fluidity of cell membranes was significantly reduced after 5 μM Aβ_1–42_ exposure, and the reduction was significantly suppressed by treatment with FA (*p* = 0.0011 vs. 5 μM Aβ_1–42_), Cur (*p* = 0.00348 vs. 5 μM Aβ_1–42_), or Cur + FA (*p* < 0.0001 vs. 5 μM Aβ_1–42_) for 30 min. Compared with 1 μM Cur alone, combination treatment with Cur and FA (*p* = 0.0396 vs. 1 μM Cur, Tukey) after exposure to Aβ_1–42_ resulted in a significant increase in cell membrane fluidity (Figure 7).

#### 2.4.2. Phospholipid Peroxidation in the Cell Membrane

**Figure 8:**
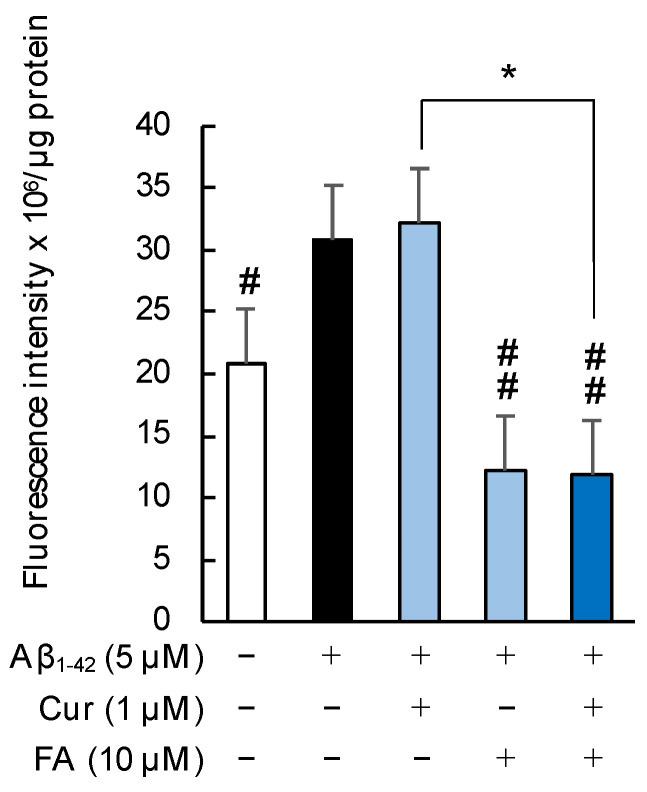
Effect of Cur, FA, or a combination of both on membrane phospholipid peroxidation level in Aβ_1–42_-stimulated SH-SY5Y cells. The levels of membrane phospholipid peroxidation in Aβ_1–42_-stimulated SH-SY5Y cells was evaluated using DPPP. The levels of membrane phospholipid peroxidation in SH-SY5Y cells exposed to 5 µM Aβ_1–42_ and treated with Aβ_1–42_ + 1 µM Cur, Aβ_1–42_ + 10 µM FA or Aβ_1–42_ + Cur + FA. In the absence of 5 μM Aβ_1–42_, the membrane phospholipid peroxidation levels of control, 1 μM Cur, 10 μM FA and Cur + FA-treated cells were 20.58 ± 2.41, 21.97 ± 2.18, 18.31 ± 1.54 and 17.77 ± 1.93 fluorescence intensity × 10^6^/μg protein (no significant difference, n = 6, Tukey). +: inclusion of 5 μM Aβ_1__–– 42_, 1 μM Cur, 10 μM FA, respectively, −: non-inclusion. The *p*-values in ANOVA were <0.001. Each value expresses the mean + S.E.M. of at least 3 independent experiments. #, *p* < 0.05; ##, *p* < 0.01 for Aβ_1–42_ exposed cells versus the other treated cells (n = 6, Tukey); *, *p* < 0.001; for Aβ_1–42_ + Cur + FA-treated cells versus Cur-treated cells (n = 6, Tukey).

As shown in Figure 8, the phospholipid peroxidation in cell membranes was significantly increased by 5 μM Aβ_1–42_ exposure, and the increase was significantly suppressed by treatment with FA (*p* = 0.0012 vs. 5 μM Aβ_1–42_) or combination of Cur + FA (*p* = 0.0009 vs. 5 μM Aβ_1–42_) for 30 min. The combination of Cur and FA significantly reduced phospholipid peroxidation levels compared to treatment with Cur alone (*p* = 0.0009, Tukey). Moreover, the combination of 5 μM Cur + 10 μM FA led to a significant reduction, compared to treatment with 5 μM Cur alone (*p* = 0.0006, Tukey) (Figure S3B).

### 2.5. Changes in Intracellular Calcium ([Ca^2+^]_i_) following Treatment with Cur, FA, or a Combination of Both

**Figure 9:**
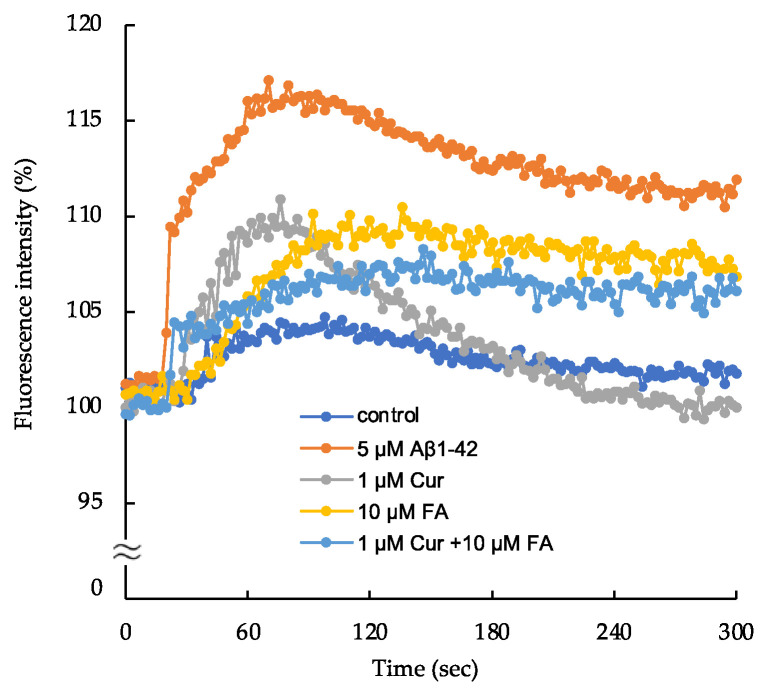
Detection of changes in intracellular ionized calcium concentration ([Ca^2+^]_i_) in SH-SY5Y cells.

As shown in Figure 9 anf Figure S4, the changes in [Ca^2+^]_i_ observed in SH-SY5Y cells increased with treatment of 5 μM Aβ_1–42_. The increase in [Ca^2+^]_i_ by 5 μM Aβ_1–42_ reached a peak at 60 s after exposure and remained elevated at an nearly constant plateau level thereafter. The peak levels were lower in cells treated with 1μM Cur, 10 μM FA or Cur + FA combination compared to cells exposed to 5 μM Aβ_1–42_ (after 60 s of exposure, Aβ_1–42_: 116.0 ± 1.19% vs. control: 103.4 ± 0.62%; *p* < 0.0001, 1 μM Cur: 108.6 ± 1.05%; *p* < 0.0001, 10 μM FA: 105.8 ± 0.71%; *p* < 0.0001, Cur + FA: 104.3 ± 1.31%; *p* < 0.0001, n = 4, Tukey). However, the peak level of [Ca^2+^]_i_ was not significantly different between Cur + FA combination and Cur (*p* = 0.5134) or FA (*p* = 0.9805) alone (n = 4, Tukey).

The plateau levels of [Ca^2+^]_i_ after 300 s of exposure were lower in cells treated with 1 μM Cur, 10 μM FA or Cur + FA combination compared to cells exposed to Aβ_1–42_ (Aβ_1–42_: 111.9± 0.93% vs control: 101.8 ± 0.49%; *p* < 0.0001, 1 μM Cur: 100.0 ± 0.88%; *p* < 0.0001, 10 μM FA: 106.8 ± 1.26%; *p* = 0.0165, Cur + FA: 106.1± 0.77%; *p* = 0.0431, Tukey, n = 4). At 300 s, the [Ca^2+^]_i_ level in cells treated with Cur + FA combination was not significant to cells treated with Cur (Tukey, n = 4, *p* = 0.2422).

Changes in [Ca^2+^]_i_ were measured for fluorescence intensity in cells exposed to 5 μM Aβ_1–42_ and cells treated with Aβ_1–42_ + Cur, Aβ_1–42_ + FA, or Aβ_1–42_ + Cur +FA. The control fluorescence intensity was added with 20 mM HEPES and 1 × Hank’s Balanced Salt solution. The fluorescence intensity was evaluated with the value at onset as 10%.

## 3. Discussion

In AD, Aβ abnormalities precede the onset of cognitive dysfunction by approximately 25 years [28]. Therefore, it is believed that starting treatment from an early stage before the onset of the disease, rather than after the onset of cognitive dysfunction, delays the onset of cognitive dysfunction. Furthermore, currently approved AD drugs, such as AChE inhibitors and NMDA receptor antagonists, constitute only symptomatic treatments with minor effects and no impact on long-term disease progression [29]. Long-term prophylactic approaches are, however, considered essential for realistic measures for AD treatment. One such practical long-term approach is the intake of readily available dietary supplements and natural products such as fruits, vegetables, and seeds. However, high daily intake of specific agents might be difficult to maintain when taken as dietary supplements. Therefore, the combined use of low doses of the targeted active ingredients may provide synergistic effects and help individuals overcome these difficulties.

In the current study, we selected Cur and FA as active ingredients for the treatment of Aβ abnormalities. Because each has a different mechanism of action, their combination may directly improve the disease state of multiplex AD. The mechanism of action of Cur on AD has been reported to involve the inhibition of Aβ aggregation, an increase in BDNF, and a decrease in tau phosphorylation [10,30,31]. On the other hand, research has shown that FA inhibits Aβ production via downregulation of APP and β-secretase [32], has potent antioxidant properties, and protects neurons from Aβ-induced neurotoxicity mainly although we previously confirmed FA also has anti-amyloidogenic effect for Aβ [21]. We hypothesized that FA and Cur might complement each other based on such reports.

As one of the features of AD pathology, Aβ accumulation in the brain causes conformational changes in peptides, forming oligomers and fibrils that deposit on plaques [5,30]. It has recently been proposed that the aggregation mechanism of Aβ peptide is that soluble Aβ monomers self-associate via conformational changes to form β-sheet-rich oligomers, and that the monomers further bind to the oligomers and elongate to form Aβ fibrils [5,33]. Here, the effect of Cur, FA and their combination on the aggregation of Aβ_1–40_ and Aβ_1–42_ peptides was monitored through temporal changes in fibrillar β-sheet content using ThT assays. Cur inhibited the aggregation of Aβ_1–42_ at low concentrations, and the inhibitory effect was dose-dependent (Figure 2B). Both Aβ_1–40_ and Aβ_1–42_ were used at 25 μM in the ThT assay. Cur showed an inhibitory effect on Aβ aggregation at a low concentration of 1 μM, indicating that Cur inhibited aggregation even at a concentration of 1/25 of that of Aβ. Cur has been reported to inhibit Aβ aggregation including oligomerization in vitro and in vivo [9,10]. Furthermore, Cur binds directly to Aβ and thereby inhibits Aβ aggregation [10]. An NMR analysis showed that Cur interacts with amino acid residues number 12 and 17–21 of Aβ_1–42_ [34], suggesting that it has an inhibitory effect on Aβ fibril elongation. The plant polyphenols myricetin, morin, and EGCG were previously reported to have an inhibitory effect on Aβ aggregation, including oligomerization. Myricetin and morin inhibit β-sheet-rich oligomer formation from soluble Aβ monomers [35]. EGCG inhibits Aβ fibril formation by promoting non-toxic oligomer formation (“off-pathway” aggregation) and inhibits Aβ fibril formation [36]. Several studies have reported that the phenolic hydroxyl group of polyphenol compounds that bind to histidine is required for anti-Aβ aggregation [37]. Cur has been shown to prevent the peptide-peptide interaction between Phe15 and His18, which is essential for Aβ aggregation [38]. Furthermore, quinones generated from phenolic hydroxyl groups react with the Lys side chains of proteins [39]. These polyphenolic compounds react with Lys28, which is essential for Aβ aggregation and may contribute to the inhibition of Aβ aggregation [40]. Moreover, quinones produced from phenolic hydroxyl groups may react with the Lys28 side chains of Aβ peptide and contribute to the inhibition of Aβ aggregation. On the other hand, in this experiment, FA had no significant effect on Aβ aggregation, but the combination of Cur and FA led to significant inhibition of aggregation of both Aβ_1–40_ and Aβ_1–42_ compared to Aβ alone at endpoint (Figure 2A,B). The combination of Cur and FA may result in a coordination with each other, such that Cur prevented the peptide-peptide interaction of Aβ and FA reacted with the Lys residue of Aβ.

Oxidative stress is normally regulated by the antioxidant defense system. However, in many diseases, that system is disrupted, and oxidative stress is known to play an essential role in pathogenesis. In particular, it leads to the accumulation of oxidative damage in AD, is associated with age-related neurodegenerative diseases, and represents the most common cause of dementia in the elderly. Aβ induces oxidative stress in vivo and in vitro and is considered an early event in AD as it contributes to membrane damage and cell death [41]. Furthermore, the extensive oxidative damage observed in brain regions with mild cognitive impairment (MCI) also suggests that oxidative stress may be an early event in the progression from normal aging to AD [42]. However, how Aβ causes oxidative stress is currently unknown. In the present study, Aβ_1–42_ increased ROS, mitochondrial-ROS, and the peroxidation of plasma membrane phospholipids in SH-SY5Y (Figure 5, Figure 6A and Figure 8). Since Aβ_1–42_ was exposed extracellularly, it is possible that Aβ_1–42_ first contacted the cell membrane and induced phospholipid peroxidation. Many studies have shown that Aβ as an oligomer inserts into the plasma membrane bilayer and initiates lipid peroxidation [43]. Aβ-induced lipid peroxidation then promotes Ca^2+^ influx into neurons, increases toxicity, and facilitates apoptosis [44]. Considering the report by Nakayama et al., demonstrating an increase in fibrils in Aβ after 3 h of incubation, it is possible that the Aβ_1–42_ used in the current experiment contained more fibrils than the oligomers [45] and that even the fibril-rich Aβ_1–42_ induced peroxidation of plasma membrane phospholipids and increased mitochondrial-ROS and [Ca^2+^]_i._ (Figure 6A, Figure 8 and Figure 9). The increase in cytoplasmic Ca^2+^ with Aβ_1–42_ exposure has been shown to cause disruption of mitochondrial homeostasis, leading to the production of ROS [46]. Mitochondrial dysfunction and oxidative stress are interacting processes. Increased oxidative stress leads to decreased glucose metabolism and ATP synthesis in the brain. Thus, oxidative stress is closely associated with Aβ neurotoxicity and plays an important role in the pathological mechanisms underlying AD. Both Cur and FA are polyphenols with antioxidant activity, and especially FA exhibits high antioxidant activity. In the current study, combined treatment with Cur and FA induced stronger antioxidant effects against Aβ_1–42_-induced oxidative stress, such as lower ROS levels, lower plasma membrane phospholipid peroxidation, and lower mitochondrial ROS levels, compared to Cur and FA treatment alone (Table 1, Figure 5, Figure 6A and Figure 8). FA also has antioxidant properties due to its phenolic hydroxyl group, while the hydroxy and phenoxy groups donate electrons to scavenge free radicals. In in vivo experiments, FA has been shown to protect against Aβ_1–42_-induced oxidative stress and neurotoxicity in primary cortical neurons in rats [47]. In vitro studies have shown that FA inhibits Aβ_1–42_-induced cell death and apoptosis in LAN5 neuroblastoma cells [48], indicating that it may be beneficial in the prevention and treatment of AD. Similarly, in the present experiments, FA treatment alone significantly inhibited Aβ_1–42_-induced oxidative stress. The o-methoxyphenyl group and methylene hydrogen in Cur contribute to the compound’s antioxidant activity, by donating electrons/hydrogen atoms to ROS and neutralizing reactive oxygen intermediates [49]. The significant antioxidant effect of Cur + FA on Aβ-induced oxidative stress may thus be the result of a complex mechanism.

In the present experiment, Aβ_1–42_ exposure reduced mitochondria-localized Mn-SOD and treatment with Cur and FA could restore the expression of Mn-SOD protein (Figure 6B). A study in vitro reported that Aβ directly interfered with mitochondrial respiration [50]. SOD is a reactive oxygen species scavenger, acting on ROS generation and protecting cells from cellular damage due to oxidative stress. However, combined treatment with Cur and FA showed no significant effect on Mn-SOD levels (Figure 6B). Furthermore, 5 μM Cur treatment eliminated the difference compared to Aβ_1–42_ exposure, and combined treatment with 5 μM Cur + 10 μM FA significantly reduced Mn-SOD level (Figure S2C). In human cancer cells, Cur has been shown to convert its antioxidant effect into an oxidant-promoting effect [49], indicating that Cur exhibits antioxidant activity in normal cells under stressful conditions and waits for the biphasic activity of the oxidant-promoting effect in cancer cells. In our cell viability experiments at 24 h incubation, the combined treatment with 5 μM Cur + 10 μM FA showed no significant effect on viability compared to Aβ_1–42_ exposure (5 μM Cur: 53.20 ± 0.62 vs 5 μM Aβ_1–42_: 50.67 ± 1.94, n = 6, Tukey). Decreased Mn-SOD levels with 5 μM Cur + 10 μM FA combination treatment may be one of the reasons for the decreased cell viability. Prolonged exposure to Cur + FA combinations at high concentrations may reduce cell viability and should be noted.

In the present experiments, Aβ_1–42_ exposure induced the disruption of membrane integrity and increased [Ca^2+^]_i_ (Figure 7, Figure 8 and Figure 9). Alterations in the functional integrity of neuronal membranes in AD may result from interactions between Aβ and the membrane. Aβ oligomers cause membrane permeation through several hypothetical mechanisms such as ion channels in cell membranes and transmembrane oligomer pore structure formation [51]. Since the Aβ used here is presumed to have a higher fibril content than the oligomers, the Aβ-induced disruption of membrane integrity shown in Figure 7 and Figure 8 may be due to Aβ-induced oxidative stress rather than Aβ-induced formation of ion channels. The generated ROS, especially the most reactive hydroxyl radicals, cause oxidative damage to both the Aβ peptide itself and the surrounding proteins and lipids. Lipid peroxidation products of cell membranes then bind to several membrane proteins, thereby altering their protein structure and function and resulting in changes in neurotoxicity [25]. Moreover, as shown in Figure 7, Aβ_1–42_ decreased membrane fluidity. Reduced membrane fluidity by Aβ accelerates the amyloidogenic processing of APP [52].

Conversely, increased membrane fluidity shifts APP cleavage processing by α-secretase to non-amyloidogenic [53]. In vivo experiments have shown that Aβ administration reduces the membrane fluidity of synaptosomes isolated from frontal and hypothalamic neurons of 3-month-old mice [54]. In the current study, we observed that treatment with Cur, FA and Cur + FA restored membrane fluidity reduced by Aβ_1–42_ (Figure 7). FA (100 μmol/L) reduced cholesterol levels in erythrocytes as well as lipid peroxidation when incubated with human erythrocytes for 24 h [55]. Cholesterol is an essential component of cell membranes, and higher membrane cholesterol levels reduce cell membrane fluidity. Depletion of cell membrane cholesterol, in contrast, results in increased membrane fluidity. One cause of the increase in cell membrane fluidity induced by FA may be that FA reduces cell membrane cholesterol levels. Cholesterol content in mammalian phospholipid bilayers is high, ranging from 20–30% in most cells and 40–50% in erythrocytes. Cur increases the membrane fluidity of liposome membranes containing less than 20% cholesterol and diffuses into the membrane, but stiffens the liposome membranes containing 40% cholesterol [56]; Cur may thus act directly on cholesterol in the liposome membrane. In the present experiments, the combined treatment with Cur + FA significantly increased fluidity compared to Cur and FA treatment alone (Figure 7). This indicates that Cur acted directly on cell membrane cholesterol in the SH-SY5Y cells, that FA reduced cell membrane cholesterol levels and that the combination of Cur + FA increased membrane fluidity through both actions.

One of the challenges of developing drugs for AD is to design drugs that improve symptoms but have few side effects. Safety is an even more critical issue in combination treatments than in single treatments, and side effects need to be considered. The acute oral lethal dose with 50% survival (LD 50) is >2000 mg/kg for Cur [57] and FA [58] when administered to mice, suggesting that both compounds are safe. Pharmacokinetics studies of oral administration of 10 g of Cur to healthy human volunteers showed a Cmax of 2.3 μg/mL (7 μM) [59]. The concentration of Cur (1 or 5 μM) used in this experiment seems to be suitable.

Considering the safety of Cur, in vivo studies indicated that Cur is poorly absorbed from the intestine when orally administered to rats in a single dose (2 g), and that plasma concentrations were below 5 μg/mL. In brief, the tissue concentration of Cur does not lead to beneficial or detrimental effects, due to insufficient absorption via the oral route, which makes it safe for oral administration. On the other hand, FA is absorbed from the stomach and small intestine, and unrestricted FA has been found in the human plasma only 10 min after oral administration of sodium ferulate: FA thus has good bioavailability [60]. Therefore, the safety of long-term Cur and FA combination treatment should be investigated in vivo.

We find that combination treatment of Cur and FA exerts a cytoprotective effect on Aβ-induced cytotoxic effects, through multiple mechanisms. These mechanisms include the suppression of Aβ aggregation and antioxidant effects, as compared to single treatment with either Cur or FA alone (Table 1). The protective effects of the combination treatment we observed were complementary and cooperative. These findings suggest that the combination of Cur and FA may provide an effective and superior strategy for the prevention and therapeutics of AD in humans.

## 4. Materials and Methods

### 4.1. Drugs and Reagents

Human amyloid β-protein (Aβ, Human, 1–42) was purchased from Peptide Institute (Osaka, Japan). DMEM Ham’s F-12 medium and all-trans retinoic acid (ATRA) were purchased from FUJIFILM Wako Pure Chemical Corporation (Osaka, Japan). Penicillin G sodium, streptomycin sulfate, amphotericin B, fetal bovine serum (FBS), Cur, and FA were obtained from Thermo Fisher Scientific K.K. (Waltham, MA, USA). Cur and FA were dissolved in dimethyl sulfoxide (DMSO) and then disbanded in medium to achieve a final concentration of DMSO to 0.1%. The other chemicals used in this experiment were the purest commercially available.

### 4.2. The Aggregation Kinetics of Aβ_1–40_ and Aβ_1–42_

The aggregation kinetics of Aβ_1–40_ and Aβ_1–42_ was measured using the SensoLyte Thioflavin T β-Amyloid (_1–40, 1–42_) Aggregation Kit (AS-72213, AS-72214; AnaSpec, Inc., Fremont, CA, USA). To measure the aggregation kinetics of Aβ_1–40_ or Aβ_1–42_ on a 96-well black microplate, 10 μL ThT (2 mM) and 5 μL Cur or FA were added to each well and mixed with 85 μL Aβ solution (29.5 μM). The final concentration of Aβ_1–40_ or Aβ_1–42_ peptide was 25 μM, Cur was 1, 5, 10 μM, and FA was 1, 10, 20, 50 μM. The ThT fluorescence signal of Aβ_1–40_ was monitored using SpectraMax i3 (Molecular Devices, Sunnyvale, CA, USA) at an excitation wavelength of 440 nm and an emission wavelength of 484 nm at 37 °C for 6 h at 15 min intervals. The ThT fluorescence intensity of Aβ_1–42_ was monitored for 2 h under the same conditions as that of Aβ_1–42_. The mixture was shaken for 5 s during the measurement to promote aggregation. The experiment was performed in triplicate.

### 4.3. Cell Culture and Drug Treatment

SH-SY5Y cells (human neuroblastoma, EC-94030304) were obtained from the European Collection of Authenticated Cell Cultures (London, UK). SH-SY5Y cells were cultured in DMEM Ham’s F-12 containing 10% FBS, penicillin G sodium, streptomycin sulfate, and amphotericin B, and maintained in a humid atmosphere of 5% CO_2_ and 95% air at 37 °C. SH-SY5Y cells were treated with 10 µM ATRA for 5 days to differentiate. Aβ_1–42_ was dissolved in DMSO and incubated at 37 °C for 24 h for self-aggregation, and then prepared to 5 μM in a DMEM Ham’s F-12 medium without fetal bovine serum (FBS). The differentiated SH-SY5Y cells were then treated with 5 μM Aβ_1–42_ containing Cur (1, 5 μM), FA (10 μM) or the combination of both lysed in DMEM / Ham’s F-12 medium. As a control, cells cultured in a medium containing 0.1% DMSO were used. All treatments were performed aseptically.

### 4.4. Detection of Viability and Cytotoxicity in SH-SY5Y Cells

#### 4.4.1. Cell Viability Assay

An MTT assay was applied to evaluate the effect of Cur, FA and the combination of both compounds on the viability of SH-SY5Y cells exposed to Aβ_1–42_. MTT assay is based on the formation of blue formazan metabolized from colorless MTT by mitochondrial dehydrogenases, which are active only in live cells. The Cell Proliferation Kit I (11465007001, Roche, Mannheim, Germany) was used according to the manufacturer’s instructions. The differentiated SH-SY5Y cell of 1.0 × 10^5^ cells/mL were seeded into 96-well collagen-coated plates and incubated at 37 °C for 24 h. First, to assess a suitable concentration of Aβ_1–42_ to induce cytotoxicity in SH-SY5Y cells, preliminary experiments were performed in which SH-SY5Y cells were exposed to Aβ_1–42_ (1, 5 and 10 µM) for 3 h. As shown in Figure 3A, the appropriate concentration of Aβ_1–42_ to induce cytotoxicity was 5 µM. Next, to investigate the protective effects of Cur, FA and the combination of both compounds on Aβ_1–42_-induced cytotoxicity, SH-SY5Y cells were treated with Aβ_1–42_ + Cur (1, 5 μM), Aβ_1–42_ + FA (10 μM), or Aβ_1–42_ + Cur + FA for 3 h. After incubation, a MTT assay was performed and the results were measured at 540 nm using a microplate reader Spectra Max i3 (Molecular Devices Co., San Jose, CA, USA).

#### 4.4.2. Calcein-AM and EthD-1 (Live/Dead) Cell Assay

Live cells and dead cells were also observed by calcein-AM and EthD-1 costaining. SH-SY5Y cells were seeded at 1.0 × 10^6^ cells/mL in 96-well collagen-coated plates and incubated at 37 °C for 24 h, then exposed to Aβ_1–42_ or treated with Aβ_1–42_ + Cur, Aβ_1–42_ + FA or Aβ_1–42_ + Cur + FA for 3 h. The treated cells were stained with 2 μM calcein-AM and 10 μM EthD-1 (Thermo Fisher Scientific K.K, Waltham, MA, USA). The green fluorescent calcein, hydrolyzed by ubiquitous intracellular esterase in the cells, depends on the number of live cells, while EthD-1 only enters cells with damaged membranes, binds to nucleic acids, and emits bright red fluorescence proportional to the number of dead cells. Using Spectra Max i3 (Molecular Devices), the green fluorescence intensity was measured at Ex: 495 nm and Em: 530 nm, and the red fluorescence intensity at Ex: 495 nm and Em: 645 nm. The morphology of individual cells was also evaluated by observation with a fluorescence microscope (BZX800; Keyence, Osaka, Japan).

### 4.5. Assay of Oxidative Stress

#### 4.5.1. Reactive Oxygen Species (ROS) Detection

To detect the effect of Aβ_1–42_ exposure on ROS production, we used a chloromethyl derivative of CM-H_2_DCFDA (Thermo Fisher Scientific K.K, Waltham, MA, USA), which is a useful indicator for ROS detection. SH-SY5Y cells were incubated at 37 °C for 24 h, then exposed to 5 μM Aβ_1–42_ and treated with Aβ_1–42_ + Cur, Aβ_1–42_ + FA, or Aβ_1–42_ + Cur + FA for 30 min. Fluorescence intensity was measured using a Spectra Max i3 (Molecular Devices) at Ex: 488 nm and Em: 525 nm. Individual oxidative stress status was assessed by observation with a fluorescence microscope (BZX800; Keyence Co., Osaka, Japan).

#### 4.5.2. Mitochondrial ROS Assay

Mitochondrial ROS is one of the major sources of intracellular ROS. To investigate mitochondrial ROS, SH-SY5Y cells (1.0 × 10^6^ cells/mL) were exposed to Aβ_1–42_ or treated with Aβ_1–42_ + Cur, Aβ_1–42_ + FA, or Aβ_1–42_ + Cur + FA for 30 min. Mitochondrial ROS in the treated SH-SY5Y cells was detected using the Mitochondrial ROS Detection Kit (701600, Cayman Chemical Company, Ann Arbor, MI, USA). Fluorescence intensity was measured using Spectra Max i3 (Molecular Devices) at Ex: 500 nm and Em: 580 nm.

#### 4.5.3. Detection of Manganese-Superoxide Dismutase (Mn-SOD)

SH-SY5Y cells were incubated at 37 °C for 24 h, after which cells were exposed to Aβ_1–42_ and treated with Cur for 24 h. The mitochondrial SOD isozyme content in cell lysates was determined and measured by ELISA using a monoclonal antibody (Human SOD2 ELISA Kit; ab178012, Abcam, Cambridge, UK). Absorbance was measured at 450 nm using Spectra Max i3 (Molecular Devices). The protein concentration of cell lysate was determined using the protein assay dye reagent (Bio-Rad Laboratories, Inc., Hercules, CA, USA).

### 4.6. Reaction to Cell Membrane

#### 4.6.1. The Fluidity of Cell Membrane

The dynamic properties of cell membranes are important because they are associated with various pathological syndromes associated with membrane fluidity. Damage to the membrane of neurons by toxic Aβ_1–42_ has been hypothesized to be a major event of neurotoxicity in AD. To understand the interaction of Aβ_1–42_ with the lipid bilayer, we measured cell membrane fluidity. The membrane fluidity of SH-SY5Y cells was measured using the lipophilic pyrene probe pyrene decanoate (PDA) of the Membrane Fluidity Kit (ab189819, Marker Gene Technologies, Inc., Eugene, OR, USA). SH-SY5Y cells at 1.0 × 10^6^ cells/mL were exposed to 5 μM Aβ_1–42_ or treated with Aβ_1–42_ + Cur, Aβ_1–42_ + FA, or Aβ_1–42_ + Cur + FA for 30 min. The treated cells were stained with PDA, which causes excimer formation by spatial interaction, and membrane fluidity was measured according to a previously described method [40]. The ratio of monomer (Em: 372 nm) to excimer (Em: 470 nm) fluorescence was measured with a Spectra Max i3 (Molecular Devices).

#### 4.6.2. Assay of Phospholipid Peroxidation in Cell Membranes

To detect the peroxidation of phospholipids in the membranes of cells, SH-SY5Y cells (1.0 × 10^6^ cells/mL) were stained with 5 µM diphenyl-1-pyrenylphosphine (DPPP; Thermo Fisher Scientific K.K, Waltham, MA, USA) in DMSO at 37 °C for 10 min, and then phospholipid peroxidation was measured according to a previously described method [61]. Briefly, the stained cells were exposed to Aβ_1–42_ or treated with Aβ_1–42_ + Cur, Aβ_1–42_ + FA, or Aβ_1–42_ + Cur + FA for 30 min. The fluorescence intensity of DPPP oxide was monitored using Spectra Max i3 (Molecular Devices) at Ex: 351 nm and Em: 380 nm. DPPP is known to react quantitatively with hydroperoxides to produce strong fluorescent DPPP oxides.

### 4.7. Detect of Changes in [Ca^2+^]_i_

To observe changes in [Ca^2+^]_i_ levels in SH-SY5Y cells, we used the FLIPR Calcium 5 Assay Kit (R8185, Molecular Devices). In brief, the differentiated SH-SY5Y cells were loaded with FLIPR reagent containing 20 mM HEPES and 1 × Hank’s Balanced Salt solution (pH 7.4) for 60 min at 37 °C in the presence of DMEM Ham’s F-12 medium. Then, 5 μM Aβ_1–42_, 1 μM Cur, 10 μM FA, or 1 μM Cur + 10 μM FA were added 20 s after the start of measurement. Change in [Ca^2+^]_i_ was monitored at an excitation wavelength of 485 nm and an emission wavelength of 525 nm at 37 °C for 300 s at 3-s intervals using Spectra Max i3 (Molecular Devices). The fluorescence intensity at onset was expressed as 100%.

### 4.8. Statistical Analysis

Each measurement was performed in triplicate. Results were expressed as mean + S.E.M. The effects of Cur, FA or Cur + FA were compared with SH-SY5Y cells exposed to 5 μM Aβ_1–42_, which was also included in other reagents, using analysis of variance (ANOVA) followed by Tukey or Dunnett’s post hoc test. A value of *p* < 0.05 was considered statistically significant for all tests.

## Figures and Tables

**Table 1:** Pharmacological activities.

Activities	Measurement Item	Cur	FA	Cur + FA
Suppression of Aβ aggregation	Aβ_1–40_, Aβ_1–42_	+++	±	+++
Antioxidant effects	ROS, mitochondrial ROS	+	++	+++
Cell membrane effects	Fluidity, DPPP	+	+++	+++
Ca^2+^ homeostasis	[Ca^2+^]_i_	+++	+	++
Neuroprotective effects	EthD, MTT	++	++	+++

+++: Strong intensity reaction, ++: medium intensity reaction, +: weak intensity reaction, ± no change.

## Data Availability

Not applicable.

## Supplementary Materials

The following supporting information can be downloaded at: https://www.mdpi.com/article/10.3390/ijms23179685/s1.

## References

[1] Alzheimer’s Association (2021). 2021 Alzheimer’s disease facts and figures. Alzheimers Dement..

[2] Chen Z., Zhong C. (2014). Oxidative stress in Alzheimer’s disease. Neurosci. Bull..

[3] Hardy J., Selkoe D.J. (2002). The amyloid hypothesis of Alzheimer’s disease: Progress and problems on the road to therapeutics. Science.

[4] Villemagne V.L., Burnham S., Bourgeat P., Brown B., Ellis K.A., Salvado O., Szoeke C., Macaulay S.L., Martins R., Maruff P. (2013). Amyloid β deposition, neurodegeneration, and cognitive decline in sporadic Alzheimer’s disease: A prospective cohort study. Lancet Neurol..

[5] Ono K., Watanabe–Nakayama T. (2021). Aggregation and structure of amyloid β–protein. Neurochem. Int..

[6] Cory H., Passarelli S., Szeto J., Tamez M., Mattei J. (2018). The Role of Polyphenols in Human Health and Food Systems: A Mini–Review. Front. Nutr..

[7] Yamada M., Ono K., Hamaguchi T., Noguchi–Shinohara M. (2015). Natural Phenolic Compounds as Therapeutic and Preventive Agents for Cerebral Amyloidosis. Adv. Exp. Med. Biol..

[8] Zhou H., Beevers C.S., Huang S. (2011). The targets of curcumin. Curr. Drug Targets.

[9] Hamaguchi T., Ono K., Murase A., Yamada M. (2009). Phenolic compounds prevent Alzheimer’s pathology through different effects on the amyloid–beta aggregation pathway. Am. J. Pathol..

[10] Ono K., Hasegawa K., Naiki H., Yamada M. (2004). Curcumin has potent anti–amyloidogenic effects for Alzheimer’s beta–amyloid fibrils in vitro. J. Neurosci. Res..

[11] Yang F., Lim G.P., Begum A.N., Ubeda O.J., Simmons M.R., Ambegaokar S.S., Chen P.P., Kayed R., Glabe C.G., Frautschy S.A. (2005). Curcumin inhibits formation of amyloid beta oligomers and fibrils, binds plaques, and reduces amyloid in vivo. J. Biol. Chem..

[12] Brondino N., Re S., Boldrini A., Cuccomarino A., Lanati N., Barale F., Politi P. (2014). Curcumin as a therapeutic agent in dementia: A mini systematic review of human studies. Sci. World J..

[13] Anand P., Kunnumakkara A.B., Newman R.A., Aggarwal B.B. (2007). Bioavailability of curcumin: Problems and promises. Mol. Pharm..

[14] Terao J., Karasawa H., Arai H., Nagao A., Suzuki T., Takama K. (1993). Peroxyl Radical Scavenging Activity of Caffeic Acid and Its Related Phenolic Compounds in Solution. Biosci. Biotechnol. Biochem..

[15] Mukhopadhyay A., Basu N., Ghatak N., Gujral P.K. (1982). Anti–inflammatory and irritant activities of curcumin analogues in rats. Agents Actions.

[16] Kawabata K., Yamamoto T., Hara A., Shimizu M., Yamada Y., Matsunaga K., Tanaka T., Mori H. (2000). Modifying effects of ferulic acid on azoxymethane–induced colon carcinogenesis in F344 rats. Cancer Lett..

[17] Neto–Neves E.M., da Silva Maia Bezerra Filho C., Dejani N.N., de Sousa D.P. (2021). Ferulic Acid and Cardiovascular Health: Therapeutic and Preventive Potential. Mini. Rev. Med. Chem..

[18] Chaikijurajai T., Tang W.H.W. (2020). Myeloperoxidase: A potential therapeutic target for coronary artery disease. Expert Opin. Ther. Targets.

[19] Chmielowski R.A., Abdelhamid D.S., Faig J.J., Petersen L.K., Gardner C.R., Uhrich K.E., Joseph L.B., Moghe P.V. (2017). Athero–inflammatory nanotherapeutics: Ferulic acid–based poly(anhydride–ester) nanoparticles attenuate foam cell formation by regulating macrophage lipogenesis and reactive oxygen species generation. Acta Biomater..

[20] Yan J.J., Jung J.S., Kim T.K., Hasan A., Hong C.W., Nam J.S., Song D.K. (2013). Protective effects of ferulic acid in amyloid precursor protein plus presenilin–1 transgenic mouse model of Alzheimer disease. Biol. Pharm. Bull..

[21] Ono K., Hirohata M., Yamada M. (2005). Ferulic acid destabilizes preformed beta–amyloid fibrils in vitro. Biochem. Biophys. Res. Commun..

[22] Mori T., Koyama N., Guillot–Sestier M.V., Tan J., Town T. (2013). Ferulic acid is a nutraceutical β–secretase modulator that improves behavioral impairment and alzheimer–like pathology in transgenic mice. PLoS ONE.

[23] Chen Y., Dong C. (2009). Abeta40 promotes neuronal cell fate in neural progenitor cells. Cell Death Differ..

[24] Shozawa H., Oguchi T., Tsuji M., Yano S., Kiuchi Y., Ono K. (2018). Supratherapeutic concentrations of cilostazol inhibits β–amyloid oligomerization in vitro. Neurosci. Lett..

[25] Ono K., Tsuji M. (2020). Protofibrils of Amyloid–β are Important Targets of a Disease–Modifying Approach for Alzheimer’s Disease. Int. J. Mol. Sci..

[26] Chang C.C., Edwald E., Veatch S., Steel D.G., Gafni A. (2018). Interactions of amyloid–β peptides on lipid bilayer studied by single molecule imaging and tracking. Biochim. Biophys. Acta Biomembr..

[27] Meker S., Chin H., Sut T.N., Cho N.J. (2018). Amyloid–β Peptide Triggers Membrane Remodeling in Supported Lipid Bilayers Depending on Their Hydrophobic Thickness. Langmuir.

[28] Bateman R.J., Xiong C., Benzinger T.L., Fagan A.M., Goate A., Fox N.C., Marcus D.S., Cairns N.J., Xie X., Blazey T.M. (2012). Clinical and biomarker changes in dominantly inherited Alzheimer’s disease. N. Engl. J. Med..

[29] Long J.M., Holtzman D.M. (2019). Alzheimer Disease: An Update on Pathobiology and Treatment Strategies. Cell.

[30] Dolai S., Shi W., Corbo C., Sun C., Averick S., Obeysekera D., Farid M., Alonso A., Banerjee P., Raja K. (2011). “Clicke” sugar–curcumin conjugate: Modulator of amyloid–β and tau peptide aggregation at ultralow concentrations. ACS Chem. Neurosci..

[31] Ono K., Li L., Takamura Y., Yoshiike Y., Zhu L., Han F., Mao X., Ikeda T., Takasaki J., Nishijo H. (2012). Phenolic compounds prevent amyloid β–protein oligomerization and synaptic dysfunction by site–specific binding. J. Biol. Chem..

[32] Nagai N., Kotani S., Mano Y., Ueno A., Ito Y., Kitaba T., Takata T., Fujii N. (2017). Ferulic Acid Suppresses Amyloid β Production in the Human Lens Epithelial Cell Stimulated with Hydrogen Peroxide. Biomed. Res. Int..

[33] Ono K. (2018). Alzheimer’s disease as oligomeropathy. Neurochem. Int..

[34] Masuda Y., Fukuchi M., Yatagawa T., Tada M., Takeda K., Irie K., Akagi K., Monobe Y., Imazawa T., Takegoshi K. (2011). Solid–state NMR analysis of interaction sites of curcumin and 42–residue amyloid β–protein fibrils. Bioorg. Med. Chem..

[35] Ono K., Yoshiike Y., Takashima A., Hasegawa K., Naiki H., Yamada M. (2003). Potent anti–amyloidogenic and fibril–destabilizing effects of polyphenols in vitro: Implications for the prevention and therapeutics of Alzheimer’s disease. J. Neurochem..

[36] Ngo S.T., Truong D.T., Tam N.M., Nguyen M.T. (2017). EGCG inhibits the oligomerization of amyloid beta (16–22) hexamer: Theoretical studies. J. Mol. Graph. Model..

[37] Przygońska K., Pacewicz M., Sadowska W., Poznański J., Bal W., Dadlez M. (2019). His6, His13, and His14 residues in Aβ 1–40 peptide significantly and specifically affect oligomeric equilibria. Sci. Rep..

[38] Nedumpully–Govindan P., Kakinen A., Pilkington E.H., Davis T.P., Chun Ke P., Ding F. (2016). Stabilizing Off–pathway Oligomers by Polyphenol Nanoassemblies for IAPP Aggregation Inhibition. Sci. Rep..

[39] Ishii T., Mori T., Tanaka T., Mizuno D., Yamaji R., Kumazawa S., Nakayama T., Akagawa M. (2008). Covalent modification of proteins by green tea polyphenol (–)–epigallocatechin–3–gallate through autoxidation. Free Radic. Biol. Med..

[40] Sato M., Murakami K., Uno M., Nakagawa Y., Katayama S., Akagi K., Masuda Y., Takegoshi K., Irie K. (2013). Site–specific inhibitory mechanism for amyloid β42 aggregation by catechol–type flavonoids targeting the Lys residues. J. Biol. Chem..

[41] Perry G., Nunomura A., Hirai K., Takeda A., Aliev G., Smith M.A. (2000). Oxidative damage in Alzheimer’s disease: The metabolic dimension. Int. J. Dev. Neurosci..

[42] Lovell M.A., Markesbery W.R. (2001). Ratio of 8–hydroxyguanine in intact DNA to free 8–hydroxyguanine is increased in Alzheimer disease ventricular cerebrospinal fluid. Arch. Neurol..

[43] Yasumoto T., Takamura Y., Tsuji M., Watanabe–Nakayama T., Imamura K., Inoue H., Nakamura S., Inoue T., Kimura A., Yano S. (2019). High molecular weight amyloid β(1–42) oligomers induce neurotoxicity via plasma membrane damage. FASEB J..

[44] Bezprozvanny I., Mattson M.P. (2008). Neuronal calcium mishandling and the pathogenesis of Alzheimer’s disease. Trends Neurosci..

[45] Watanabe–Nakayama T., Ono K., Itami M., Takahashi R., Teplow D.B., Yamada M. (2016). High–speed atomic force microscopy reveals structural dynamics of amyloid β1–42 aggregates. Proc. Natl. Acad. Sci. USA.

[46] Kimura A.M., Tsuji M., Yasumoto T., Mori Y., Oguchi T., Tsuji Y., Umino M., Umino A., Nishikawa T., Nakamura S. (2021). Myricetin prevents high molecular weight Aβ(1–42) oligomer–induced neurotoxicity through antioxidant effects in cell membranes and mitochondria. Free Radic. Biol. Med..

[47] Sultana R., Ravagna A., Mohmmad–Abdul H., Calabrese V., Butterfield D.A. (2005). Ferulic acid ethyl ester protects neurons against amyloid beta– peptide(1–42)–induced oxidative stress and neurotoxicity: Relationship to antioxidant activity. J. Neurochem..

[48] Picone P., Bondi M.L., Montana G., Bruno A., Pitarresi G., Giammona G., Di Carlo M. (2009). Ferulic acid inhibits oxidative stress and cell death induced by Ab oligomers: Improved delivery by solid lipid nanoparticles. Free Radic. Res..

[49] Sarkar A., De R., Mukhopadhyay A.K. (2016). Curcumin as a potential therapeutic candidate for Helicobacter pylori associated diseases. World J. Gastroenterol..

[50] Casley C.S., Canevari L., Land J.M., Clark J.B., Sharpe M.A. (2002). Beta–amyloid inhibits integrated mitochondrial respiration and key enzyme activities. J. Neurochem..

[51] Butterfield S.M., Lashuel H.A. (2010). Amyloidogenic protein–membrane interactions: Mechanistic insight from model systems. Angew. Chem. Int. Ed. Engl..

[52] Peters I., Igbavboa U., Schütt T., Haidari S., Hartig U., Rosello X., Böttner S., Copanaki E., Deller T., Kögel D. (2009). The interaction of beta–amyloid protein with cellular membranes stimulates its own production. Biochim. Biophys. Acta.

[53] Yang X., Sun G.Y., Eckert G.P., Lee J.C. (2014). Cellular membrane fluidity in amyloid precursor protein processing. Mol. Neurobiol..

[54] Li Y., Wang J.J., Cai J.X. (2007). Aniracetam restores the effects of amyloid–beta protein or ageing on membrane fluidity and intracellular calcium concentration in mice synaptosomes. J. Neural Transm..

[55] Piotr D., Broncel M., Podsędek A., Koter–Michalak M. (2012). Hypolipidemic and antioxidant effects of hydroxycinnamic acids, quercetin, and cyanidin 3–glucoside in hypercholesterolemic erythrocytes (in vitro study). Eur. J. Nutr..

[56] Leite N.B., Martins D.B., Fazani V.E., Vieira M.R., Dos Santos Cabrera M.P. (2018). Cholesterol modulates curcumin partitioning and membrane effects. Biochim. Biophys. Acta Biomembr..

[57] Srimal R.C., Dhawan B.N. (1973). Pharmacology of diferuloyl methane (curcumin), a non–steroidal anti–inflammatory agent. J. Pharm. Pharmacol..

[58] Paiva L.B., Goldbeck R., Santos W.D., Squina F.M. (2013). Ferulic acid and derivatives: Molecules with potential application in the pharmaceutical field. Braz. J. Pharm. Sci..

[59] Vareed S.K., Kakarala M., Ruffin M.T., Crowell J.A., Normolle D.P., Djuric Z., Brenner D.E. (2008). Pharmacokinetics of curcumin conjugate metabolites in healthy human subjects. Cancer Epidemiol. Biomark. Prev..

[60] Yang C., Tian Y., Zhang Z., Xu F., Chen Y. (2007). High–performance liquid chromatography–electrospray ionization mass spectrometry determination of sodium ferulate in human plasma. J. Pharm. Biomed. Anal..

[61] Nagata M., Tsuji M., Oguchi T., Momma Y., Nohara T., Ohashi H., Ito N., Yamamoto K., Udaka Y., Sasaki A. (2021). Protective Effects of the Alga Fucoidan Against Amyloid–β–Induced Neurotoxicity in SH–SY5Y Cells. BPB Rep..

