# Supplementary material for: Combined Treatment with Curcumin and Ferulic Acid Suppressed the Aβ-Induced Neurotoxicity More than Curcumin and Ferulic Acid Alone
Source: Int J Mol Sci. 2022 Aug 26;23(17):9685. doi: 10.3390/ijms23179685 (PMC9456505; doi:10.3390/ijms23179685)
Supplement: Supplementary file 1 [file ijms-23-09685-s001.zip › ijms-1796846-supplementary.pdf]

International Journal of *Molecular Sciences*

MDPI

Suppl. Table S1. The p–values for all pairwise comparisons using the Tukey test for $A\beta_{1-40}$ aggregation assay

	$A\beta_{1-40}$	1 μM Cur	5 μM Cur	10 μM Cur	1 μM FA	10 μM FA	20 μM FA	50 μM FA	1 μM Cur + 10 μM FA	5 μM Cur +10 μM FA
$A\beta_{1-40}$		1.0000	**< 0.0001**	**< 0.0001**	0.9196	0.8446	0.3851	0.7101	0.0884	**< 0.0001**
1 μM Cur			**< 0.0001**	**<0.0001**	0.7232	0.523	0.1348	0.3925	0.1242	**< 0.0001**
5 μM Cur				0.5175	**< 0.0001**	**< 0.0001**	**< 0.0001**	**< 0.0001**	**0.0004**	0.9875
10 μM Cur					**< 0.0001**	**< 0.0001**	**< 0.0001**	**< 0.0001**	**< 0.0001**	0.9788
1 μM FA						1.0000	0.9997	1.0000	**0.0072**	**< 0.0001**
10 μM FA							0.9969	1.0000	**0.0008**	**< 0.0001**
20 μM FA								1.0000	**< 0.0001**	**< 0.0001**
50 μM FA									**0.0008**	**< 0.0001**
1 μM Cur + 10 μM FA										**< 0.0001**
5 μM Cur + 10 μM FA										

Thioflavin T (ThT) fluorescence assay was used to compare the effects of Cur, FA, and the combination of both on the aggregation rate of $A\beta_{1-40}$ peptide at 360 min. $A\beta_{1-40}$ is aggregation of the $A\beta_{1-40}$ peptide alone. Statistical significances are in boldface.

Suppl. Table S2. The p–values for all pairwise comparisons using the Tukey test for $A\beta_{1-42}$ aggregation assay

	$A\beta_{1-42}$	1 μM Cur	5 μM Cur	10 μM Cur	1 μM FA	10 μM FA	20 μM FA	50 μM FA	1 μM Cur + 10 μM FA	5 μM Cur + 10 μM FA
$A\beta_{1-42}$		**0.0004**	**< 0.0001**	**< 0.0001**	0.9821	0.9884	0.5242	0.572	**< 0.0001**	**< 0.0001**
1 μM Cur			**< 0.0001**	**< 0.0001**	**< 0.0001**	**< 0.0001**	**< 0.0001**	**< 0.0001**	0.9121	**< 0.0001**
5 μM Cur				**< 0.0001**	**< 0.0001**	**< 0.0001**	**< 0.0001**	**< 0.0001**	**< 0.0001**	0.0842
10 μM Cur					**< 0.0001**	**< 0.0001**	**< 0.0001**	**< 0.0001**	**< 0.0001**	**< 0.0001**
1 μM FA						1.0000	0.9988	0.9997	**< 0.0001**	**< 0.0001**
10 μM FA							0.9821	0.9911	**< 0.0001**	**< 0.0001**
20 μM FA								1.0000	**< 0.0001**	**< 0.0001**
50 μM FA									**< 0.0001**	**< 0.0001**
1 μM Cur + 10 μM FA										**< 0.0001**
5 μM Cur + 10 μM FA										

Thioflavin T (ThT) fluorescence assay was used to compare the effects of Cur, FA, and the combination of both on the aggregation rate of $A\beta_{1-42}$ peptide at 120 min. $A\beta_{1-42}$ is aggregation of the $A\beta_{1-42}$ peptide alone. Statistical significances are in boldface.

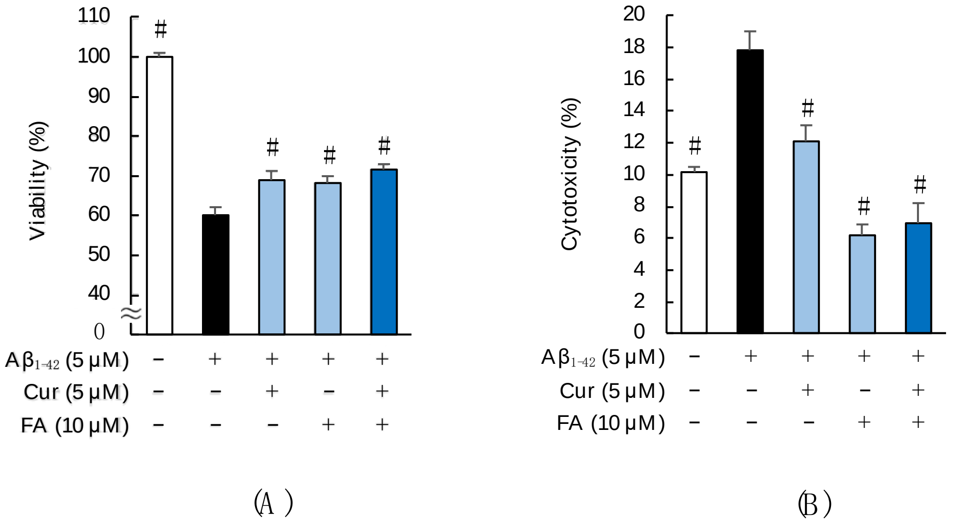

Suppl. Figure S1. Effect of Cur, FA, or a combination of both on the viability and cytotoxicity in $A\beta_{1-42}$–stimulated SH–SY5Y cells.
(A) The viability in $A\beta_{1-42}$–stimulated SH–SY5Y cells was evaluated using MTT assay. Cell viability of SH–SY5Y cells exposed with 5 μM $A\beta_{1-42}$ and treated with $A\beta_{1-42}$ + 5 μM Cur, $A\beta_{1-42}$ + 10 μM FA or $A\beta_{1-42}$ + Cur + FA for 3 hr.
(B) The cytotoxicity in $A\beta_{1-42}$–stimulated SH–SY5Y cells was evaluated using EthD–1 Cell assay. The cytotoxicity of SH–SY5Y cells exposed with 5 μM $A\beta_{1-42}$ and treated with $A\beta_{1-42}$ + 5 μM Cur, $A\beta_{1-42}$ + 10 μM FA or $A\beta_{1-42}$ + Cur + FA for 3 hr.
+: inclusion of 5 μM $A\beta_{1-42}$, 1 μM Cur, 10 μM FA, respectively, –: non–inclusion. The p–values in ANOVA were < 0.001. Each value expresses the mean + S.E.M. of at least 3 in-dependent experiments.
#, $p < 0.01$; $A\beta_{1-42}$ exposed cells versus the other treated cells (n = 6, Tukey)

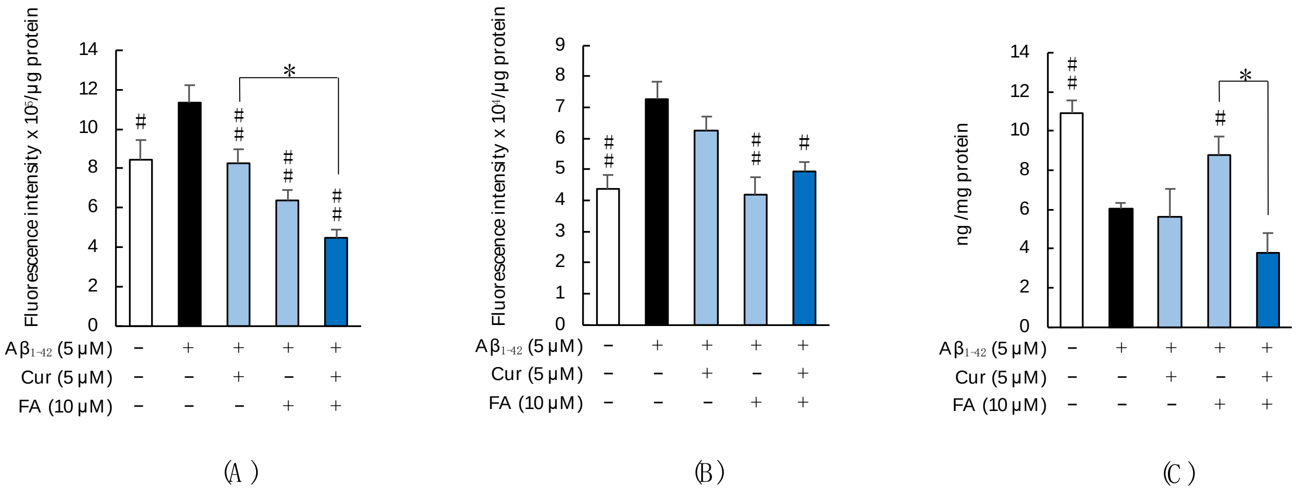

Suppl. Figure S2. Effect of Cur, FA, or a combination of both on ROS generation, mitochondrial ROS and Mn–SOD in $A\beta_{1-42}$–stimulated SH–SY5Y cells.
The generation of ROS (A), mitochondrial ROS level (B), Mn–SOD levels (C) in SH–SY5Y cells exposed with 5 μM $A\beta_{1-42}$ and treated with $A\beta_{1-42}$ + 5 μM Cur, $A\beta_{1-42}$ + 10 μM FA or $A\beta_{1-42}$ + Cur + FA.
+: inclusion of 5 μM $A\beta_{1-42}$, 1 μM Cur, 10 μM FA, respectively, –: non–inclusion. The p–values in ANOVA were < 0.001. Each value expresses the mean + S.E.M. of at least 3 in-dependent experiments.
#, $p < 0.05$; ##, $p < 0.01$ for $A\beta_{1-42}$ exposed cells versus the other treated cells (n = 6, Tukey); *, $p < 0.01$ for $A\beta_{1-42}$ + Cur + FA–treated cells versus Cur or FA–treated cells (n = 6, Tukey).

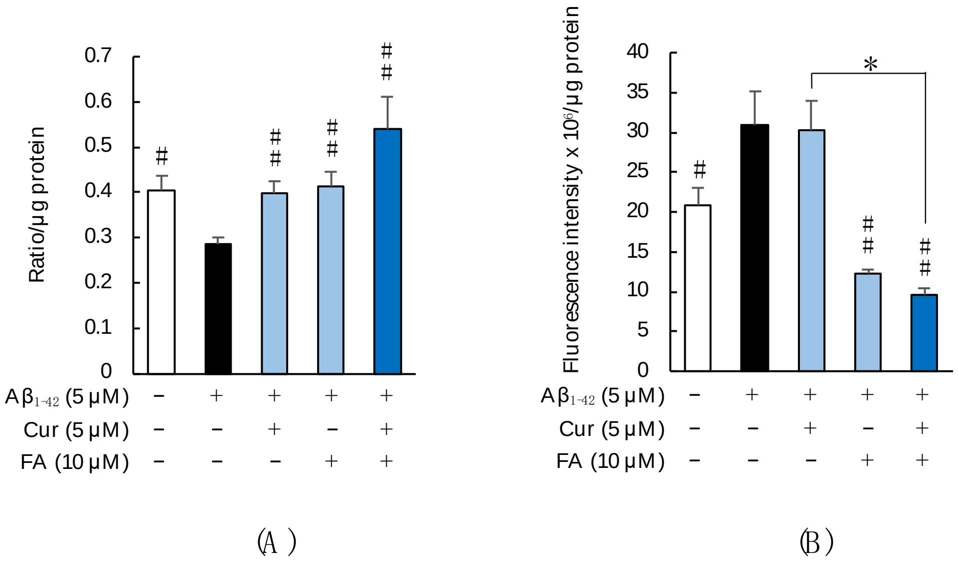

Suppl. Figure S3. Effect of Cur, FA, or a combination of both on membrane integrity in Aβ1-42–stimulated SH–SY5Y cells. The fluidity (A) and phospholipid peroxidation (B) of cell membranes in SH–SY5Y cells exposed with 5 μM Aβ1-42 and treated with Aβ1-42 + 5 μM Cur, Aβ1-42 + 10 μM FA or Aβ1-42 + Cur + FA.
+: inclusion of 5 μM Aβ1-42, 1 μM Cur, 10 μM FA, respectively, –: non–inclusion. The p–values in ANOVA were < 0.001. Each value expresses the mean + S.E.M. of at least 3 in-dependent experiments.
#, $p < 0.05$; ##, $p < 0.01$ for Aβ1-42 exposed cells versus the other treated cells (n = 6, Tukey); *, $p < 0.001$ for Aβ1-42 + Cur + FA–treated cells versus Cur–treated cells (n = 6, Tukey).

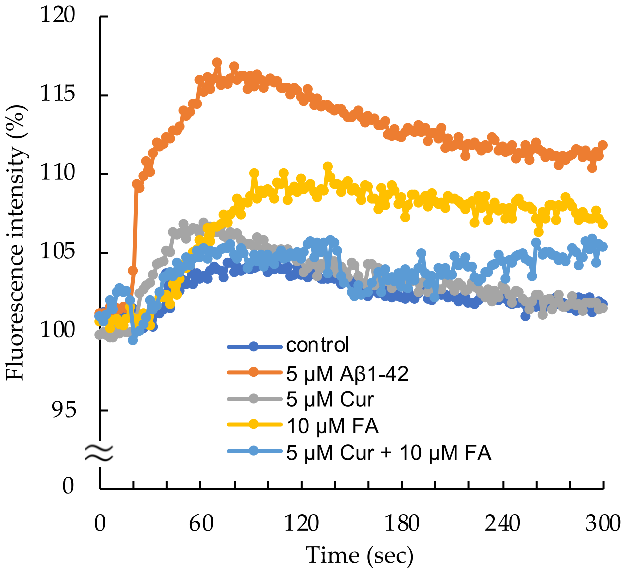

Suppl. Figure S4. Detection of changes in intracellular ionized calcium concentration ($[Ca^{2+}]_i$) in SH–SY5Y cells. Changes in $[Ca^{2+}]_i$ were measured for fluorescence intensity in cells exposed to 5 μM Aβ1-42 and cells treated with Aβ1-42 + 5 μM Cur, Aβ1-42 + FA, or Aβ1-42 + 5 μM Cur + FA. The control fluorescence intensity added with 20 mM HEPES and 1 × Hank's Balanced Salt solution. The fluorescence intensity was evaluated with the value at on–set as 100 %.
